# Single-cell mitophagy patterns within the tumor microenvironment modulate intercellular communication, impacting the progression and prognosis of hepatocellular carcinoma

**DOI:** 10.3389/fimmu.2024.1448878

**Published:** 2025-01-06

**Authors:** Zhengyan Li, Wei Chen, Shu Yao, Zuxiang Peng, Hongming Liu, Yongliang Tang, Yi Feng

**Affiliations:** Department of Hepatobiliary Surgery, Daping Hospital, Army Medical University, Chongqing, China

**Keywords:** hepatocellular carcinoma, mitophagy, tumor microenvironment, bioinformatics, prognosis

## Abstract

**Background:**

Hepatocellular carcinoma (HCC) is a common malignant tumor of the digestive system with a high incidence that seriously threatens patients’ lives and health. However, with the rise and application of new treatments, such as immunotherapy, there are still some restrictions in the treatment and diagnosis of HCC, and the therapeutic effects on patients are not ideal.

**Methods:**

Two single-cell RNA sequencing (scRNA-seq) datasets from HCC patients, encompassing 25,189 cells, were analyzed in the study. We utilized non-negative matrix factorization (NMF) clustering to identify mitophagy patterns in HCC TME cells, including cancer-associated fibroblasts (CAFs), T cells, B cells, and tumor-associated macrophages (TAMs). Cell-to-cell communication was analyzed using the CellChat package, and pseudotime trajectory analysis was performed using the Monocle package. Gene regulatory networks were investigated with the SCENIC package, and survival analyses were conducted with mitophagy-related signatures.

**Results:**

HCC samples analysis identified 22 clusters, including 7 principal cell types. Complex cell communications were observed among these cell types. Mitophagy-related CAFs, TAMs, CD8+ T cells, and B cells were identified. These subtypes had different biological states, cell-cell communications, and metabolic pathways. Mitophagy levels were elevated in tumor samples. Changes in mitophagy-related genes within specific cell subtypes were associated with different overall survival rates. However, mitophagy did not seem to affect the effectiveness of immunotherapy.

**Conclusion:**

This study provides evidence that mitophagy within the HCC TME modulates intercellular communication, influencing tumor progression and patient prognosis. Targeting mitophagy may offer a promising approach to improve the long-term prognosis of HCC patients.

## 1 Introduction

Hepatocellular carcinoma (HCC), a common malignant tumor of the digestive system, is the leading cause of cancer-related death, and its incidence is rapidly increasing (1). Early surgical resection (2) in combination with targeted therapy and immunotherapy (3, 4) is the main treatment option for HCC. These standardized treatments greatly improve the survival rate of patients with HCC. However, HCC has a high rate of drug resistance (5), recurrence, and metastasis (6), which greatly influences patient survival. Therefore, identifying novel biomarkers and gaining a deeper understanding of their molecular mechanisms may provide new insights into the diagnosis and treatment strategies for HCC, contribute to risk stratification, and ultimately optimize the treatment and prognosis of HCC.

Healthy mitochondria are intracellular energy factories that not only produce energy (ATP) through oxidative phosphorylation (7) but also are involved in other cellular functions (8). In contrast, malfunctioning mitochondria generate excessive reactive oxygen species (ROS) (9), which may harm essential cellular components, such as DNA. Mitophagy, a form of selective autophagy, preserves mitochondrial function and cellular homeostasis by specifically eliminating dysfunctional mitochondria from the cytoplasm (10, 11). To maintain mitochondrial homeostasis after mitochondrial damage, mitophagy must be precisely controlled and balanced by new mitochondrial biogenesis (11). The accumulation of mitochondrial defects due to inadequate mitophagy may cause a variety of diseases (10), including cancer (12). Dysregulated mitophagy results in the regeneration of healthy mitochondria, leading to the accumulation of defective mitochondria, which has been linked to various pathological conditions.

HCC exhibits a notably intricate tumor microenvironment (TME) (13), with a variety of infiltrating cell types, including fibroblasts (14), malignant mesenchymal tumor cells (15), intricate vascular networks (16), and immune cells (17). Evidence indicates that TME significantly contributes to tumor development, metastasis, and drug resistance (18). Moreover, single-cell transcriptomics has revealed complex intercellular communication among distinct subtypes of TME cells in cancers. TME cells include cancer-associated fibroblasts (CAFs), T cells, B cells, and tumor-associated macrophages (TAMs) (19, 20). Complex interactions between different tumor cell types and the TME have been reported to facilitate cancer progression at multiple levels. The underlying mechanisms of action of these TME cells in HCC are not fully understood. Hence, understanding the specific mechanisms and interactions between cell clusters and the TME can provide insights into HCC and guide diagnostic and therapeutic strategies.

In our study, we investigated the impact of mitophagy on major HCC TME cells (including CAFs, T cells, TAMs, and B cells) using single-cell RNA sequencing (scRNA-seq) and delved deeper into the functional implications of mitophagy across various cell types within the TME. We analyzed various patterns of mitophagy HCC TME cells using non-negative matrix factorization (NMF) clustering based on the properties of mitophagy regulators. We found that cells with mitophagy patterns had different biological states and cell–cell communications. Transcriptional property correlations, metabolic pathways, Kyoto Encyclopedia of Genes and Genomes (KEGG) enrichment analysis, and the overall prognosis were analyzed. We identified key pathways that are differentially regulated by mitophagy in TME cells, offering insights into potential therapeutic targets. Furthermore, our study highlights the prognostic significance of mitophagy in HCC and demonstrates its potential as a biomarker of disease outcome. By linking mitophagy-related alterations with patient survival data, we provided evidence that mitophagy plays a pivotal role in modulating the immune landscape of the TME, thereby influencing tumor progression and patient prognosis.

## 2 Materials and methods

### 2.1 Experimental design and data collection

We sourced two scRNA-seq datasets from patients with HCC from GSE166635 in the Gene Expression Omnibus (GEO) database (https://www.ncbi.nlm.nih.gov/geo). Samples were collected from the tumors and adjacent tissues of two patients with HCC. After sample integration, we performed batch correction procedures and acquired whole matrix data comprising gene expression and phenotypes spanning 25,189 cells in total. The obtained matrix was used as the basis for subsequent scRNA analysis. Subsequently, clinical information and bulk mRNA sequencing data for 869 patients with HCC were sourced from the UCSC Xena [cohort: GDC TCGA Liver Cancer (LIHC)] and GEO databases (GSE54236). After data correction, the obtained matrix was used as the test and verification group for subsequent analyses. In this study, all the data were sourced from previously published outlets or publicly accessible repositories.

### 2.2 Cell types of visualization and subtypes clustering within HCC samples

The Seurat package was used to construct an scRNA-seq matrix. We first created Seurat objects and set the filter threshold as follows: cells should express less than 4,000 but more than 500 genes, and mitochondrial counts should be less than 20%. Cells that do not meet the criteria are considered of low quality and are filtered out. The top 2,000 genes were selected as the most highly variable features. All these data were deemed the research foundation, and we then used the Seurat package’s FindVariableFeatures function. Next, we utilized the ScaleData and RunPCA functions to determine the number of principal components, and 1:20 was set as the threshold for the RunPCA functions. The “t-SNE” and “UMAP” techniques were chosen for further dimensionality reduction. Subsequently, we annotated cell types to infer the information from previous studies (21) within HCC; these cell types included macrophages (*CD163* and *CD68*); epithelial cells (*EPCAM* and *CCDH1*); CAFs (*AACTA2* and *COL1A2*); T cells (*CD3D*, *CD8A*, and *CCD4*); dendritic cells (*ITGAX*); endothelial cells (*PECAM1*); NK cells (*FGFBP2*); and B cells (*CCD19*, *CD79A*, and *JCHAIN*). We used this information for further cell annotation and visualization of the HCC samples.

### 2.3 Cell-to-cell communication analysis

The CellChat package was used for the cell-to-cell communication analysis to explore potential interactions. First, we constructed a CellChat object using scRNA data processed by NMF. Subsequently, the CellChat database was used to investigate cell communication and identify ligand–receptor pairs within these cell types. These interactions were then utilized to discern specific communication patterns. In addition, we computed the probability of communication between cells to further investigate the molecular interaction networks among various cell types, including dendritic cells, CAFs, macrophages, endothelial cells, B cells, epithelial cells, and T cells. In particular, we focused on communication between tumor endothelial cells and TME subtypes characterized by mitophagy-related genes.

### 2.4 NMF of mitophagy-associated genes in HCC TME cells

To further investigate the effects of mitophagy on different TME cell types, we performed a dimensionality reduction analysis to study the expression patterns of 29 mitophagy related genes in the major HCC TME cell types. We further filtered the scRNA data and performed dimensionality-reduction clustering. Subsequently, a NMF algorithm that provides a sparse representation of the data helps identify underlying patterns or structures with fewer components, which we used to identify different cell subtypes in these TME cell populations.

### 2.5 Regulation of mitophagy gene for HCC TME cells in pseudotime trajectory analysis

To further analyze cell pseudotime trajectories and mitophagy regulators, we utilized the Monocle package to analyze distinct NMF cell types in HCC. Initially, we identified highly variable genes based on the criteria of mean expression levels ≥ 0.1 and empirical dispersion ≥ 1 * dispersion fit. Next, we utilized the “plot pseudotime heatmap” function to create heatmaps illustrating the dynamic expression patterns of mitophagy regulators along the pseudotime trajectories of different TME cell types in HCC.

### 2.6 Marker gene identification for mitophagy-related genes within HCC TME cell subtypes

After isolating the target cell clusters, we utilized the FindAllMarkers function to identify marker genes for NMF clusters within the HCC cell types. We specified a threshold log fold change (logFC) of 0.5 and prioritized genes with a logFC greater than 1, focusing on the mitophagy-related genes that ranked highest in the list. Next, the NMF clusters were renamed according to these genes. To visualize the results, the DotPlot function was used to display the top-ranking genes with the highest expression levels in each NMF cluster. Additionally, a feature plot function was used to illustrate the distribution of specific mitophagy genes in the TME of HCC.

### 2.7 Functional enrichment analysis of NMF mitophagy-related subtypes

After mitophagy clusters were identified across various TME cell types, we employed the clusterProfiler R package to investigate the Reactome pathway databases, genomes, and KEGG based on these marker genes. The CytoScape enrichment map function was used to visualize and organize the pathways. We considered gene sets with an adjusted *p*-value of less than 0.05 as significantly enriched. The top three pathways associated with these mitophagy clusters were prioritized.

### 2.8 SCENIC analysis of NMF mitophagy-related subtypes

We used the aertslab/SCENIC package obtained from GitHub to investigate the gene regulatory network involving transcription factors (TFs) in HCC. Two gene-motif rankings, specifically hg19-tss-centered-10 kb and hg19-500 bp-upstream, sourced from the RcisTarget database, were employed to facilitate the identification of transcription start sites and explore gene regulatory networks within the scRNA-seq data of HCC. Subsequently, TFs with adjusted *p*-values less than 0.05 corrected using the Benjamini–Hochberg method were selected for further investigation in subsequent analyses.

### 2.9 Survival analyses with mitophagy-related signatures in mRNA-sequence datasets

Initially, we used the ggplot package to visualize mitophagy levels between normal and tumor groups. Further analysis was then conducted to examine mitophagy abundance. Next, we generated mitophagy-related gene signatures for all HCC NMF cell clusters using the FindAllMarkers function. Concurrently, the predominant cell types within the HCC TME were determined based on the scRNA-seq data. Subsequently, the GSVA function was employed to compute the scores of these gene signatures across publicly available HCC datasets. To investigate the correlation between mitophagy-related NMF signatures and patient outcomes, including overall survival rates, we performed the log-rank test and Cox proportional hazard regression. The cutoff values for various NMF cell signatures in diverse public datasets were determined using the Survminer R package, enabling the generation of Kaplan–Meier survival curves. To obtain comprehensive prognostic information from NMF mitophagy-related signatures across multiple public datasets, we applied the RMA function from the meta for R package to merge the Cox regression results for identical signatures. Finally, the forestplot R package was used to visually depict the meta-analysis results.

### 2.10 Prediction of immunotherapy analysis

We acquired mRNA data and survival information for HCC samples from both the UCSC Xena and GEO databases. Following initial filtering, these datasets were uploaded to the Tumor Immune Dysfunction and Exclusion (TIDE) website to predict the response to immunotherapy in patients with HCC. Additionally, we explored the association between mitophagy-related NMF signatures by analyzing the output data. Moreover, the immune checkpoints obtained from public datasets were evaluated against their respective datasets.

## 3 Results

### 3.1 Single-cell visualization of HCC samples

**Figure 1:**
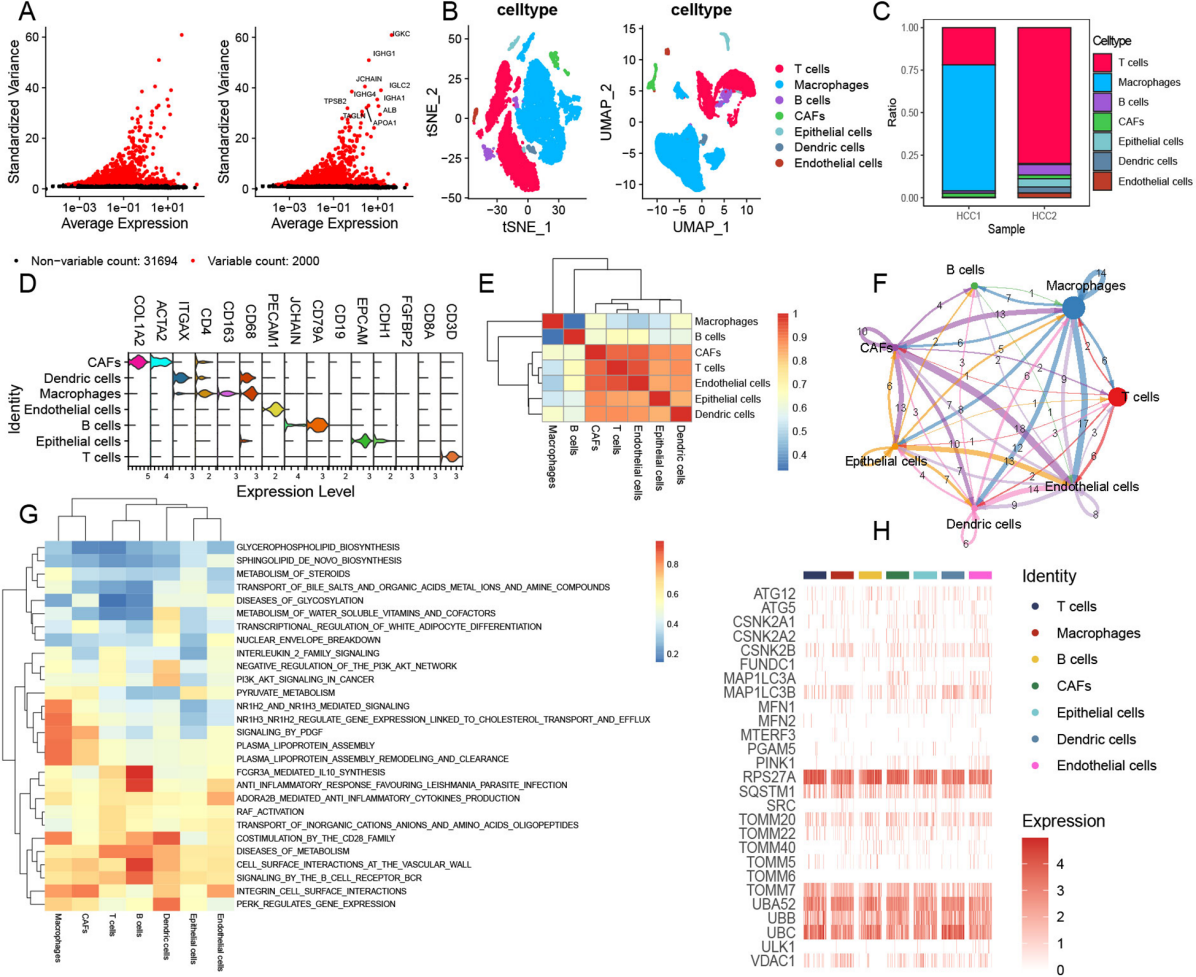
Comprehensive landscape of mitophagy-related genes in single-cell HCC data. **(A)** Top 10 variables expressing features in HCC data. **(B)** tSNE and UMAP plots showing the main cell types of HCC. **(C)** Cell composition within each HCC sample. **(D)** Annotated marker genes for major cell types. **(E)** Heatmap showing the correlation of main cell types. **(F)** Cell communications among seven primary cell types via CellChat analysis. **(G)** GSVA enrichment analysis of main cell types. **(H)** Heatmap illustrating the distribution of mitophagy-related genes across major cell types.

To explore the TME and cell diversity within the HCC, HCC samples from two patients were analyzed. After quality control (**Figure 1A**), 33,694 cells were obtained. We performed UMAP and tSNE analyses to further investigate the cell composition and cellular landscape of HCC, ultimately identifying 22 clusters (**Supplementary Figures 1A, B**). Referring to previous studies, we annotated each cluster and identified seven principal clusters: T cells, macrophages, B cells, CAFs, epithelial cells, endothelial cells, and dendritic cells (**Figure 1B**). The proportion of different cell types in each sample was also analyzed (**Figure 1C**). T cells and macrophages had the largest cell populations. Next, we visualized the markers (**Figure 1D**) and analyzed the correlation between these cell types (**Figure 1E**). Strong relationships were observed among CAFs, T cells, epithelial cells, dendritic cells, and endothelial cells.

We performed CellChat analysis to identify complex cell communications among these cell types in HCC samples (**Figure 1F**). CAFs exhibit complex cellular interactions with other cell types, particularly HCC endothelial cells and macrophages. At the same time, we also found that epithelial cells and macrophages have rich signal transduction in the whole cell communication. MIF and MDK pathways were significantly enhanced in CAFs compared to other cells (**Supplementary Figure 1C**). In addition, endothelial cells showed remarkable communication with other cells, and signals from endothelial cells showed weakening of MDK (**Supplementary Figure 1C**). The ligand–receptor pairs included but not limited to MDK–(ITGA4+ITGB1), MDK–(ITGA6+ITGB1), MDK–LRP1, MDK–NCL, MDK–SDC1, MDK–SDC2, MDK–SDC4, MIF–(CD74+CD44), MIF–(CD74+CXCR4), and MIF–ACKR3 were enriched. The transduction patterns of these signals may contribute to tumor invasion and metastasis. We also performed GSVA enrichment for further analysis; these seven cell types showed obvious pathway-activated states (**Figure 1G**). We further evaluated the expression of mitophagy-related genes in these HCC clusters (**Figure 1H**) and found a significant abundance of mitophagy-related regulators such as RPS27A, TOOMM7, UBB, and UBC within these clusters. The mitophagy expression model revealed intrinsic similarities among the seven HCC tumor cell types. Notably, RPS27A encodes components of the small ribosomal subunit, which is involved in protein synthesis. It is also associated with the ubiquitin–proteasome system (UPS), playing a key role in maintaining mitochondrial quality control and promoting mitochondrial autophagy through the degradation of damaged proteins. TOMM7 is a constituent of the translocase of the outer mitochondrial membrane complex, responsible for importing proteins into the mitochondria. Furthermore, TOMM7 is involved in regulating the PINK1/Parkin pathway, which is essential for the identification and clearance of damaged mitochondria. UBB and UBC encode ubiquitin, a protein critical for tagging damaged proteins and mitochondria, thereby targeting them for degradation via the UPS and autophagy pathways.

### 3.2 Mitophagy-related CAFs contributed to the HCC TME

**Figure 2:**
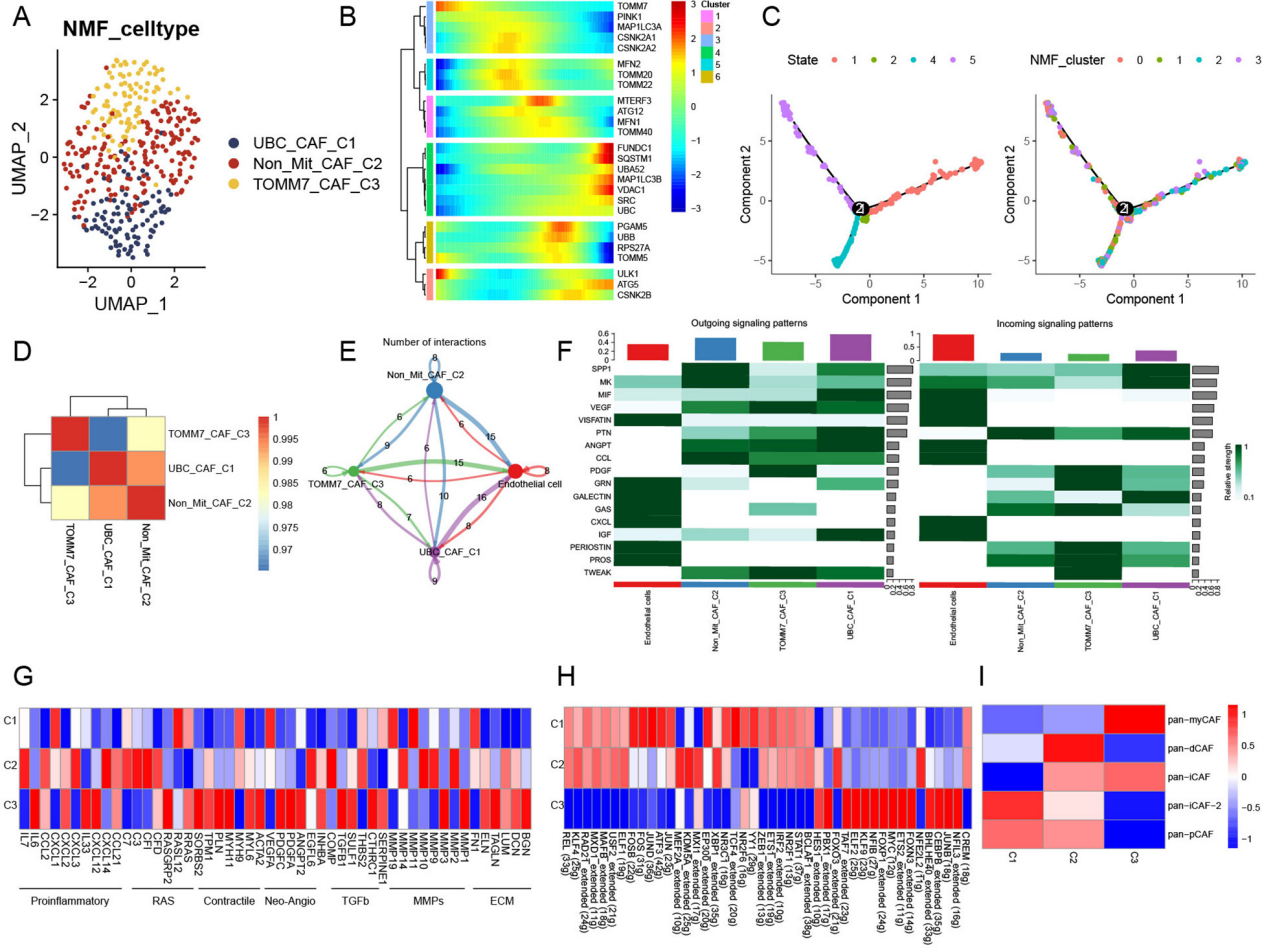
Novel regulation patterns of mitophagy-related CAFs. **(A)** UMAP plot showing mitophagy-related NMF subtypes of CAFs. **(B)** The trajectory analysis of the mitophagy-related gene dynamics in CAFs. **(C)** Pseudotime analysis of CAF mitophagy-related NMF clusters. **(D)** Heatmap showing the correlation of NMF subtypes of CAFs. **(E)** Cell–cell communications among mitophagy-related CAFs and endothelial cells. **(F)** Relative strength of signal pathways among mitophagy-related CAFs and endothelial cells. **(G)** Heatmap of different average expression of common signaling pathway genes in the three NMF CAF subtypes. **(H)** Heatmap showing differential TF activities among three mitophagy-related CAFs. **(I)** Relationship between CAFs subtypes and aggrephagy-related CAFs.

CAFs were selected aside, and quality control checks were conducted by constructing a mitophagy-related expression matrix and removing cells that did not express mitophagy-related genes. Subsequently, a dimensionality reduction was performed (**Supplementary Figure 2A**). We identified four new CAF subtypes after NMF treatment. We analyzed the abundance of mitophagy-related genes and refined these four clusters to obtain UBC-CAF-C1, Non-Mit-CAF-C2, and TOMM7-CAF-C3 cells (**Figure 2A**). Pseudo-time analysis revealed the crucial involvement of mitophagy-related genes in the trajectory of CAFs, with a predominant role observed during the intermediate and advanced stages of CAF differentiation (**Figures 2B, C**). Simultaneously, we found that UBC-CAF-C1 and Non-Mit-CAF-C2 were strongly correlated (**Figure 2D**). Following the analysis of cell–cell communication, we observed that the mitophagy-related CAF clusters exhibited varying numbers of ligand–receptor interactions compared to endothelial cells, and ligand–receptor links involving endothelial cells were also very strong (**Figure 2E**). In addition, the signaling pathways exhibited the most negative stimulation, whereas MIF signaling pathway activation was observed in cell communication (**Supplementary Figure 2B**). Mitophagy-related CAFs showed the most enhanced signaling pathway, with the MIF signaling pathway appearing to be the main communication pathway between CAFs and endothelial cells (**Figure 2F**).

Non-Mit-CAF-C2 and TOMM7-CAF-C3 cells showed markedly different expression patterns for these pathway genes in the pathway heatmap (**Figure 2G**). Most of the pathway genes in TOMM7-CAF-C3 cells were upregulated, whereas those in UBC-CAF-C1 cells were downregulated. The expression levels of 44 TFs among the three clusters showed significant differences in gene regulatory network analysis. Notably, the TFs NFIL3, JUNB, CEBPB, BHLHE40, FOXN3, ETS2, MYC, FOXP1, NFIB, KLF9, ELF2, TAF7, FOXO3, PBX1, and HES1 were upregulated in the TOMM7-CAF-C3 cluster, but downregulated in the UBC-CAF-C1 and Non-Mit-CAF-C2 groups. The other TFs exhibited the opposite behavior (**Figure 2H**). Furthermore, we observed alterations in the metabolic pathways of mitophagy-related CAFs (**Supplementary Figure 2C**), which may be attributed to changes in mitophagy. Additionally, we computed pan-CAF signatures that have been previously reported to significantly affect tumors. Pan-myCAF is characterized by high α-SMA expression and myoblast-like properties, displaying strong contractility. They influence tumor stiffness through mechanical forces and promote invasion and metastasis by secreting specific extracellular matrix (ECM) components. Pan-dCAFs secrete numerous cytokines and chemokines that regulate the TME to enhance tumor cell proliferation, invasion, and metastasis. Pan-iCAFs are linked to inflammation and secrete high levels of proinflammatory cytokines, such as IL-6, IL-8, and CXCL12, which activate inflammatory responses, promote tumor malignancy, and suppress effective antitumor immunity. Pan-pCAFs directly support tumor growth and progression by promoting angiogenesis, regulating tumor cell proliferation and survival, and increasing resistance to therapy by interacting with tumor cells and other TME components. Our analysis revealed that the Non-Mit-CAF-C2 score was positively correlated with nearly all CAF subtypes. However, the scores of the mitophagy-related subtypes UBC-CAF-C1 and TOMM7-CAF-C3 negatively correlated with those of nearly all CAF subtypes. Specifically, the UBC-CAF-C1 was associated with the pan-immunosuppressive CAF (pan-iCAF) subtype, whereas the TOMM7-CAF-C3 score was strongly associated with the pan-myCAF subtype (**Figure 2G**).

### 3.3 Mitophagy-related TAMs resembled classical features

**Figure 3:**
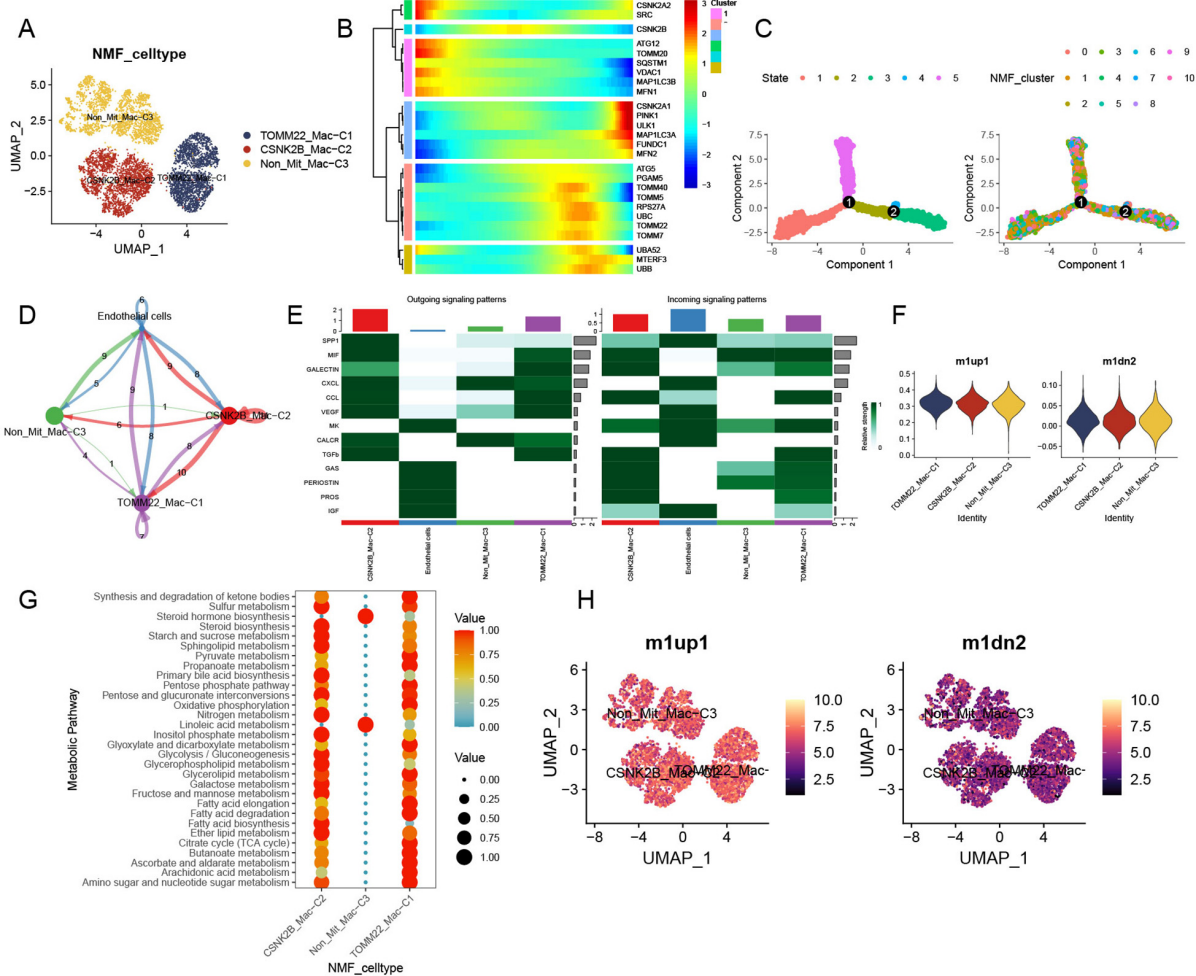
Classical features and unique contribution of mitophagy-related TAMs. **(A)** UMAP plot of mitophagy-related subtypes of NMF TAMs. **(B)** Trajectory analysis of mitophagy-related genes in TAMs. **(C)** Pseudotime analysis of mitophagy-related TAM NMF clusters. **(D)** Cell communications among mitophagy-related TAMs and tumor endothelial cells. **(E)** Relative strength of enriched outgoing and incoming signals among mitophagy-related TAMs and endothelial cells. **(F)** M1 and M2 activity assessment of mitophagy-related TAMs. **(G)** Top 30 metabolic pathway enrichment of mitophagy-related TAM subtypes. **(H)** UMAP plot of mitophagy-related NMF TAM subtypes displaying M1 and M2 activity.

In total, 8,277 tumor macrophages were isolated from the HCC macrophage dataset, which initially contained 10,323 cells (**Supplementary Figure 3A**). Subsequently, 11 macrophage clusters were identified after quality control and NMF processing (**Supplementary Figure 3B**) and were ultimately integrated into the TOMM22-Mac-C1, CSNK2B-Mac-C2, and Non-Mit-Mac-C3 clusters (**Figure 3A**). In pseudotime analysis, mitophagy-related genes were involved in both the early and late stages of TAM differentiation (**Figures 3B, C**). Additionally, we observed varying numbers of ligand–receptor interactions between these mitophagy-related macrophage clusters and endothelial cells, similar to observations in HCC CAFs (**Figure 3D**). TOMM22-Mac-C1 and CSNK2B-Mac-C2 cells exhibited more pronounced cellular communication compared to Non-Mit-Mac-C3, with the activation state of the signaling pathways further confirming these notable differences (**Figure 3E**). Specifically, the SPP1 and VEGF signaling pathways showed remarkable activation. Additionally, we conducted a macrophage metabolism analysis to further evaluate the association between mitophagy-related macrophage clusters and specific pathways. Thirty metabolic pathway enrichment results were obtained (**Figure 3G**). Enhanced metabolism, including glycolysis/gluconeogenesis, sulfur metabolism, and fatty acid biosynthesis, has been observed in mitophagy-related macrophage clusters. The AddModuleScore function was used to assess these signatures across all macrophages. Our analysis revealed that Non-Mit-Mac-C3 were significantly associated with anti-inflammatory macrophages, whereas TOMM22-Mac-C1 and CSNK2B-Mac-C were significantly associated with proinflammatory macrophages (**Figures 3F, H**).

### 3.4 Contribution of mitophagy-related CD8+ T cell to TME of HCC

**Figure 4:**
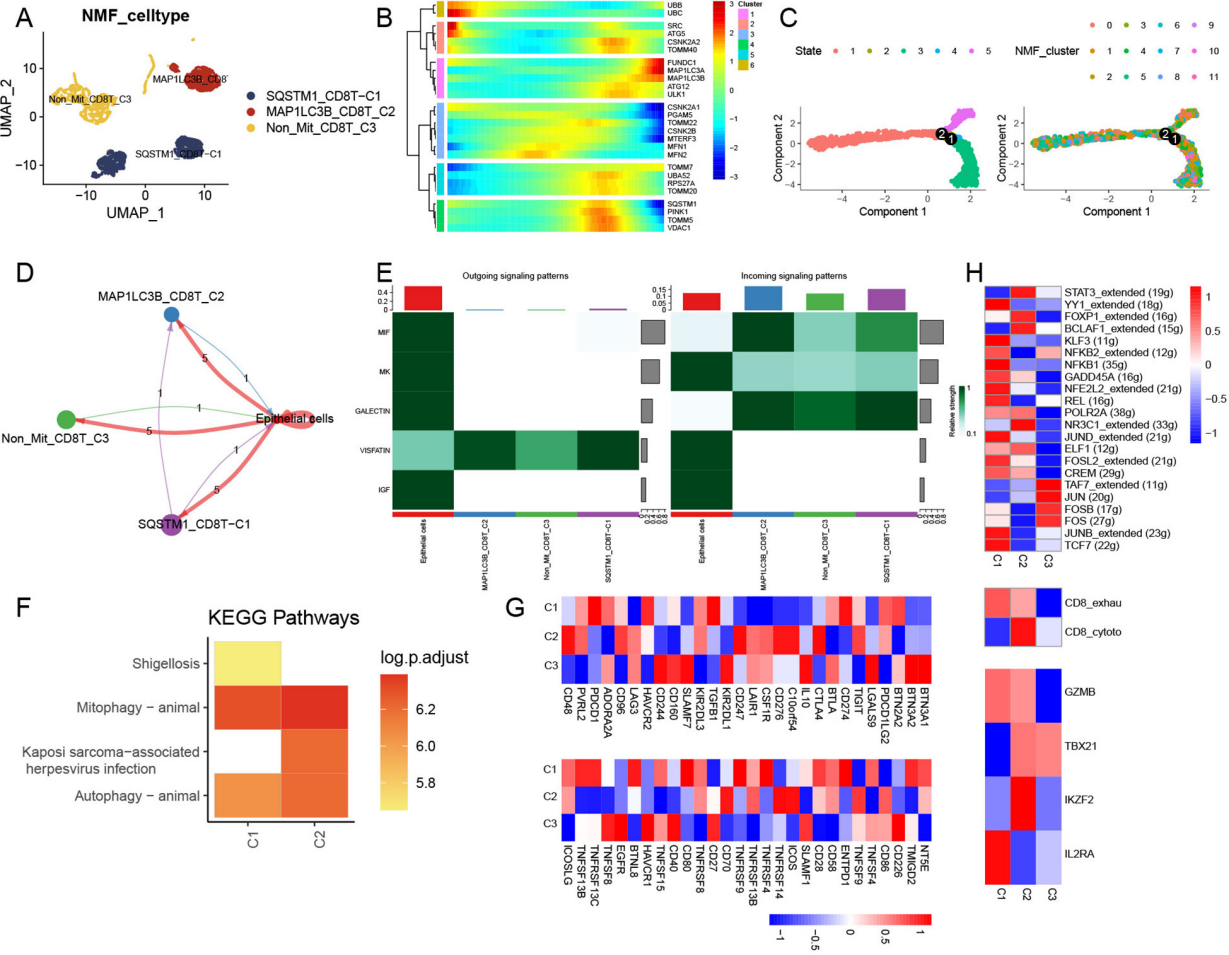
Regulation of CD8+ T NMF mitophagy-related clusters. **(A)** UMAP plot showing the landscape of mitophagy-related NMF CD8+ T cells. **(B)** Trajectory analysis of mitophagy-related genes in CD8+ T cells. **(C)** Pseudotime analysis of NMF clusters in CD8+ T cells. **(D)** Cell–cell communications among mitophagy-related CD8+ T-cell subtypes and endothelial cells. **(E)** Relative strength of enriched signal patterns among CD8+ T-cell subtypes and endothelial cells. **(F)** Heatmap illustrating activated KEGG pathways in mitophagy-related CD8+ T cells. **(G)** Heatmap displaying diverse features among mitophagy-related CD8+ T-cell clusters, immune stimulators, and immune inhibitors. **(H)** Heatmap showing differential activities of TFs, CD8+ T-cell subtypes, and T-function markers among three mitophagy-related CD8+ T-cell clusters.

Among the 6,342 T cells detected, CD8+ and CD4+ cells were identified (**Supplementary Figure 3C**). We selected CD8+ T cells as the basis for the following analysis. CD8+ T cells were subsequently divided into 12 clusters (**Supplementary Figure 3D**) and integrated into three mitophagy-related CD8+ T cell subtypes, including SQSTM1-CD8T-C1, MAP1LC3-CD8T-C2, and Non-Mit-CD8T-C3 (**Figure 4A**). We found that mitophagy-related genes mainly participated in the late stages of cell differentiation (**Figures 4B, C**). Ligand–receptor links between these mitophagy-related CD8+ T-cell clusters and endothelial cells were also observed (**Figure 4D**); there was no communication between SQSTM1-CD8T-C1 and Non-Mit-CD8T-C3, MAP1LC3-CD8T-C2, and Non-Mit-CD8T-C3, and epithelial cells were the main signal senders (**Figure 4D**). The VISFATIN signaling pathway showed remarkable activation in the outgoing and incoming signaling patterns among these cell types (**Figure 4E**). KEGG enrichment analysis showed that the SQSTM1-CD8T-C1 and MAP1LC3-CD8T-C2 clusters exhibited mitophagy and autophagy animal pathway activation; the Non-Mit-CD8T-C3 clusters did not demonstrate remarkable pathway enrichment (**Figure 4F**). Furthermore, to evaluate the collective impact of mitophagy-related CD8+ T-cell clusters on T cells, we observed numerous differences in the mean expression levels of immune genes related to co-stimulation, co-inhibition, and certain function-associated markers (**Figure 4G**). Network regulatory analysis revealed significant differential expression of TFs and T-cell markers in the mitophagy-related clusters SQSTM1-CD8T-C1 and MAP1LC3-CD8T-C2. Interestingly, SQSTM1-CD8T-C1 cells exhibited markedly upregulated TFs (**Figure 4H**). Furthermore, we observed significant differences in the average expression of signatures among the mitophagy clusters of CD8+ T cells, including T exhaustion and T cytotoxic scores. Notably, the Non-Mit-CD8T-C3 score demonstrated a contrasting trend compared to the other mitophagy clusters (**Figure 4H**).

### 3.5 Effect of mitophagy−mediated B-cell phenotypes in the TME of HCC

**Figure 5:**
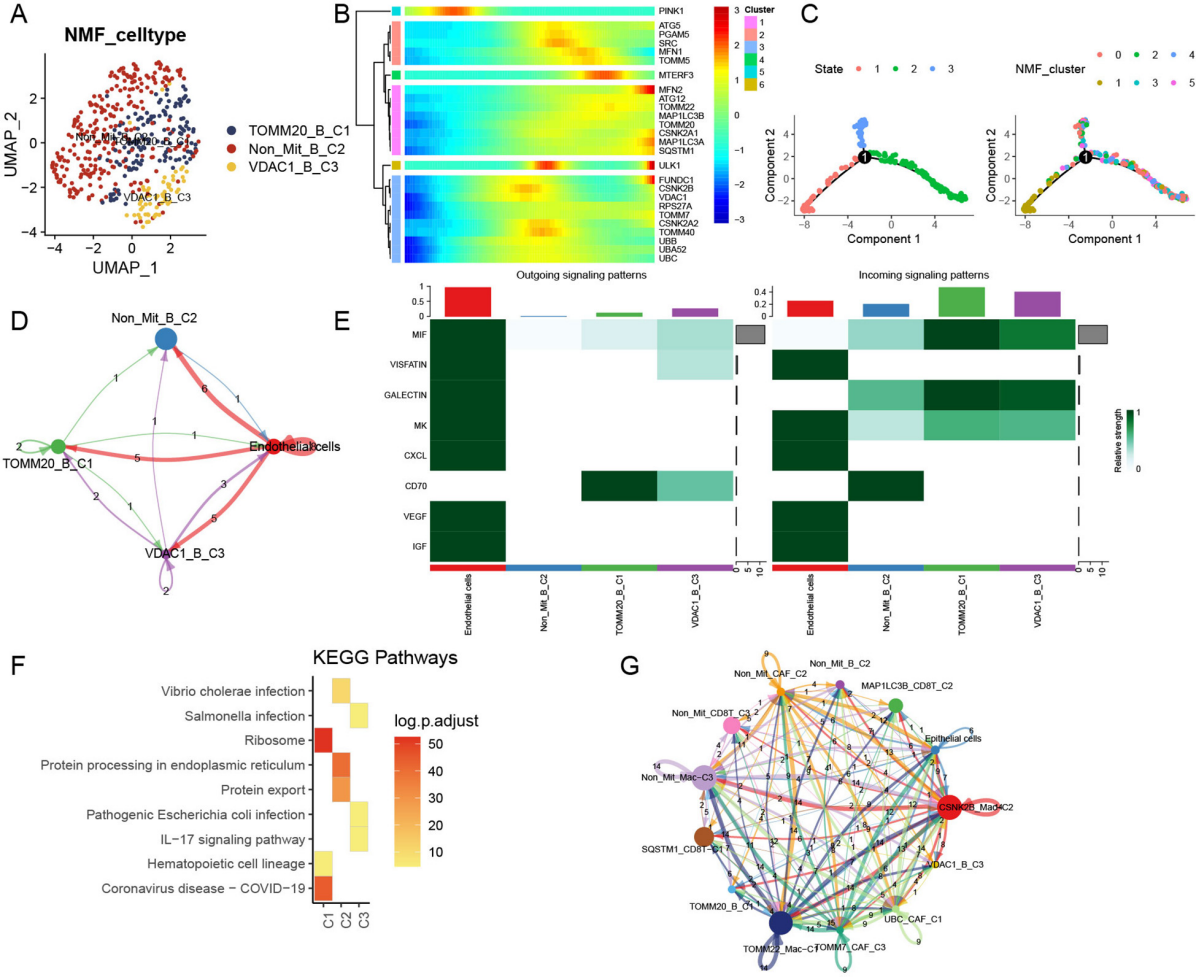
Influence of mitophagy-related B-cell subtypes in TME. **(A)** UMAP plot depicting mitophagy-related subtypes of NMF B cells. **(B)** Trajectory analysis of mitophagy-related genes in NMF B-cell subtypes. **(C)** Pseudotime analysis of B-cell subtypes. **(D)** Cell–cell communication models among mitophagy-related B-cell subtypes to endothelial cells. **(E)** Relative strength of enriched signals (outgoing and incoming) among mitophagy-related B-cell subtypes and endothelial cells. **(F)** Heatmap illustrating activated KEGG pathways in mitophagy-related B-cell subtypes. **(G)** Cell–cell communications among all mitophagy-related subtypes and endothelial cells.

We extracted a total of 450 B cells from the scRNA data and integrated them into four clusters (**Supplementary Figures 3E, F**). After NMF clustering, TOMM20-B-C1, Non-Mit-B-C2, and VDAC1-B-C3 cells were obtained (**Figure 5A**). Mitophagy-related genes were also involved in the middle and late stages of B-cell development (**Figures 5B, C**). The number of ligand–receptor connections between mitophagy-related B-cell clusters and endothelial cells varied. Similar to CD8+ T cells, the main communication interactions between these clusters mainly originate from HCC endothelial cells (**Figure 5D**). Clusters related to mitophagy emitted more signals than other clusters (**Figure 5E**). Additionally, KEGG analysis revealed that TOMM20-B-C1 significantly activated the ribosomal pathway, VDAC1-B-C3 significantly activated the IL-17 signaling pathway, and Non-Mit-B-C2 significantly activated protein processing in the endoplasmic reticulum (**Figure 5F**). After integrating all mitophagy-related clusters and performing further analysis, we found that these clusters showed complex cell communication and that B-cell subtypes play roles in receivers (**Figure 5G**).

### 3.6 Mitophagy-related TME patterns guide HCC prognosis and immunotherapy

**Figure 6:**
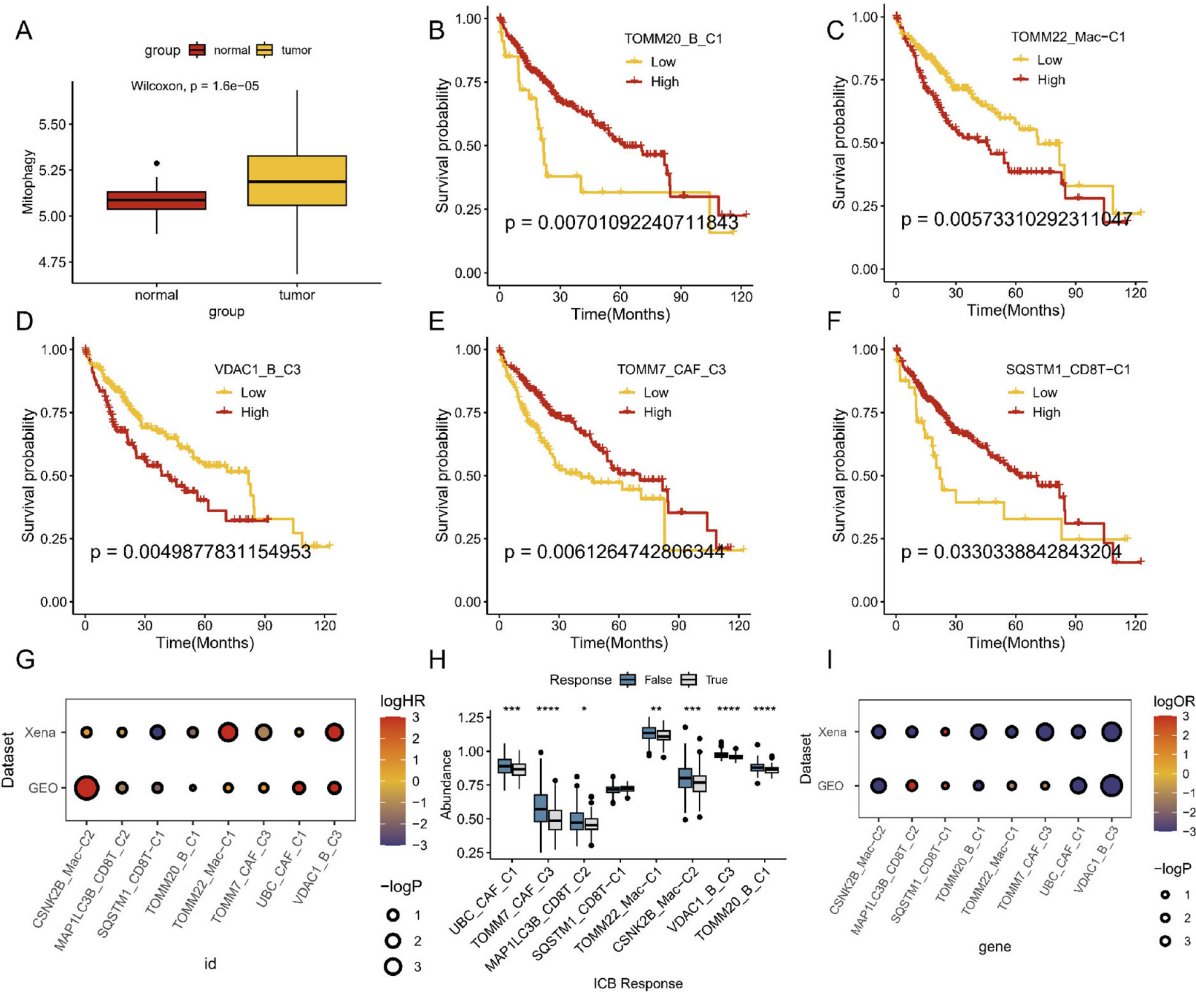
Prognosis and immunotherapy response of mitophagy-related cell types in bulk sequencing. **(A)** Whole mitophagy level between normal and tumor in mRNA sequencing. **(B–F)** Survival analyses of different mitophagy-related TME cell subtypes. **(G)** Overall survival analysis across different datasets. **(H)** ICB efficiency analyses of different mitophagy-related TME cell subtypes. **(I)** ICB analysis across different datasets.

To further explore the mitophagy levels in HCC, we tested mitophagy levels and abundance, analyzed the signature of the main HCC TME-related cell types, and recalculated mitophagy-related gene expression in the bulk data. We found that mitophagy levels were significantly elevated and varied in abundance in tumor samples compared to normal tissues (**Figure 6A**). Based on these findings, we utilized the GSVA function to compute mitophagy subscores and examined their prognostic implications in patients with HCC. Subsequently, we performed Cox regression analysis to identify the cell subtypes that were significantly associated with survival rate and further visualized the survival curves. Remarkably, changes in mitophagy-related genes within specific related genes within specific cell subtypes, such as CAFs, macrophages, CD8+ T cells, and B cells, were linked to significantly different overall survival rates among these subclusters (**Figures 6B–F**). We further validated these findings using GEO data and observed similar results (**Figure 6G**). Additionally, we used the TIDE website for pre-analysis and identified transcriptomic biomarkers in patients with HCC to predict their treatment responses (**Figure 6H**). Findings from the UCSC Xena dataset support these results, demonstrating outcomes similar to those observed in the GEO dataset (**Figure 6I**). However, it is worth noting that the beneficial effect of current immune-based therapies beneficial for patients with cancer may not effectively target individuals with high mitophagy-related HCC.

### 3.7 Mitophagy-mediated TME patterns enhanced the intercellular communication

All ligand–receptor pairs of intercellular communication were subsequently listed by comprehensive cell chat analysis of all HCC cells. These ligand–receptor pairs, which existed from mitophagy subclusters to the tumor endothelial cells, included the following: DM–CALCRL, ANGPT1–TEK, ANGPT2–(ITGA5+ITGB1), ANGPT2–TEK, CCL2–ACKR1, CCL3–CCR1, CCL3L3–CCR1, CCL5–ACKR1, CCL5–CCR1, CD70–CD27, CXCL2–ACKR1, CXCL3–ACKR1, CXCL8–ACKR1, GAS6–AXL, GAS6–MERTK, GRN–SORT1, GZMA–F2R, GZMA–F2RL3, IGF1–(ITGA6+ITGB4), IGF2–(ITGA6+ITGB4), IGF2–IGF2R, LGALS9–CD44, LGALS9–CD45, LGALS9–HAVCR2, MDK–(ITGA4+ITGB1), MDK–(ITGA6+ITGB1), MDK–LRP1, MDK–NCL, MDK–SDC1, MDK–SDC2, MIF–(CD74+CD44), MIF–(CD74+CXCR4), MIF–ACKR3, NAMPT–(ITGA5+ITGB1), NAMPT–INSR, PDGFA–PDGFRB, PGF–VEGFR1, POSTN–(ITGAV+ITGB5), PROS1–AXL, PTN–NCL, PTN–SDC1, PTN–SDC2, PTN–SDC3, RARRES2–CMKLR1, SPP1–(ITGA4+ITGB1), SPP1–(ITGA5+ITGB1), SPP1–(ITGAV+ITGB1), SPP1–(ITGAV+ITGB5), SPP1–CD44, TGFB1–(TGFBR1+TGFBR2), TGFB3–(TGFBR1+TGFBR2), TNF–TNFRSF1A, TNF–TNFRSF1B, TNFSF12–TNFRSF12A, TNFSF13B–TNFRSF13B, TNFSF13B–TNFRSF13C, TNFSF13B–TNFRSF17, VEGFA–VEGFR1, VEGFA–VEGFR1R2, VEGFA–VEGFR2, VEGFB–VEGFR1, and VEGFC–VEGFR2 (**Supplementary Figure 4**). Different mitophagy subtypes may have distinct strengths and ligand–receptor interactions with tumor cells, indicating that mitophagy-mediated TME cells might interact more extensively with tumor cells, thereby contributing to HCC progression.

## 4 Discussion

Mitophagy is essential for maintaining mitochondrial function and homeostasis. Insufficient mitophagy can lead to the accumulation of mitochondrial defects, which is a major cause of various diseases (22). Understanding the precise mechanisms of mitophagy in tumors is crucial for advancing drug research and treatment strategies.

While many studies have explored the link between mitophagy and HCC pathogenesis (23, 24), few have examined this association at the single-cell level. In this study, we are the first to comprehensively explore mitophagy-associated genes across major cell types in the HCC TME. We also identified diverse patterns of cell–cell communication between mitophagy-related TME subtypes and tumor cells, with a particular focus on endothelial cells. This new perspective enhances our understanding of how mitophagy in different cellular components of the TME impacts clinical outcomes in patients with HCC.

Cancer endothelial cells play pivotal roles in tumor growth, metastasis, invasion, immune evasion, and therapeutic responses (25). An increasing number of studies have underscored the significance of communication between endothelial cells and cells within the TME. The diversity of this intercellular communication affects patient responsiveness to treatment and prognostic outcomes (26). In our study, we observed diverse regulatory patterns of mitophagy in TME cells, along with extensive interactions with tumor endothelial cells. Additionally, cell-chat analysis revealed ligand–receptor pairs and the activation of angiogenesis-related pathways, elucidating the involvement of mitophagy-related cell subtypes in angiogenesis.

Within the intricate TME, our analysis revealed that CAFs engage in intricate cellular communication with endothelial cells. Notably, CAFs exhibited significantly heightened outgoing signaling patterns, particularly activating pathways involving VEGF, ANGTP, and IGF. These findings strongly suggest that CAFs are involved in tumor angiogenesis, thereby contributing to the rapid establishment of neovascular networks within the TME (27). HCC always shows significant vascular infiltration, which often leads to a poor prognosis (28). Previous studies have reported that CAFs play a vital role in the progression of angiogenesis in tumors (28). Interestingly, our results suggest that mitophagy-associated CAFs play a central role in tumor angiogenesis. They have been shown to activate the VEGF signaling pathway and produce copious amounts of proangiogenic factors such as VEGF, thereby actively fostering neovascular network expansion within the TME. In addition, endothelial cell activation plays a significant role in the building blocks of the tumor vascular network (6). In our prognostic analysis, high levels of mitophagy-related CAFs were associated with improved survival in patients with HCC. We observed that Non-Mit-CAF-C2 exhibited activation of the SPP1 signaling pathway, consistent with previous reports that SPP1+TAMs compromise tumor immunity and have a proangiogenic profile (29). In contrast, mitophagy-related CAFs displayed reduced SPP1 expression. The improved survival rates observed in patients with high mitophagy-related CAFs are likely due to the regulation of both the SPP1 and VEGF signaling pathways, which enhance their overall effectiveness in the TME.

In the traditional classification, CAFs can be further categorized as pan-dCAFs, pan-nCAFs, pan-iCAFs, pan-pCAFs, and pan-myCAFs based on distinct molecular characteristics (30). In our investigation, we unveiled the potential involvement of mitophagy in CAFs. Furthermore, Non-Mit-CAF-C2 and TOMM7-CAF-C3 had a strong relationship with inflammatory CAFs, but the expression of proinflammatory factors was not typical in TOMM7-CAF-C3, possibly because of the weakened expression of most inflammatory factors as a result of cell mitophagy (31). This may also explain the high survival rate of TOMM7-CAF-C3 cells. Furthermore, our findings indicated that non-mitophagy-related CAFs exhibited high expression levels of TGFb and ECM inflammatory factors. These factors likely promote ECM degradation and distant tumor metastasis (32), potentially contributing to the unfavorable prognosis associated with non-mitophagic CAFs. Consequently, we hypothesized that mitophagy-associated CAFs attenuate immunosuppressive interactions with tumor cells, thereby restricting tumor progression and metastasis.

In recent years, research has increasingly focused on the crucial role of immune cell regulation and reprogramming in tumors, particularly in TAMs (33). In this study, we identified a significant phenomenon in which mitophagy-associated macrophages exhibit extensive intercellular communication with tumor endothelial cells. Our CellChat analysis revealed that the CSNK2B+Mac and TOMM22+Mac subtypes demonstrated activation of the SPP1, MIF, and CCL signaling pathways. Subsequent prognostic analyses revealed a positive correlation between their expression levels and survival probability. In contrast, mitophagy-related CAFs cells showed activated SPP1 signaling patterns. This phenomenon may also arise from the potential attenuation of inflammatory factor expression in CAFs due to mitophagy. Metabolic processes, including glucose, glutamine, and fatty acid metabolism, exert a significant influence on TAMs in modulating not only cancer progression but also immune responses (34). Our investigation revealed that mitophagy-related macrophage subtypes exhibited remarkable activation of metabolic pathways, such as oxidative phosphorylation and the citrate cycle (TCA cycle). These metabolic changes cause TAMs to reprogram toward M1-type polarization (35, 36). However, such polarization did not result in improved patient survival, possibly due to the activation of more VEGF in the TGFb pathway by mitophagy-associated macrophage subpopulations. In addition, a study found that SPP1 expression is elevated in immune cells, particularly in inflammatory states, and may be involved in immune and inflammatory processes by regulating oxidative phosphorylation (37). This may also explain poor survival rates. It is not difficult to see that patients may benefit from interventions targeting the metabolic pathways corresponding to different mitophagy subtypes.

Furthermore, apart from macrophages, we observed that mitophagy-associated CD8+ T and B cells exhibited similar patterns of cellular communication, as they did not demonstrate intricate interactions with tumor cells. Endothelial cells are the main senders and receivers of cellular communication. Interestingly, we observed the activation of the VISFATIN signaling pathway in CD8+ T-cell communication. This pathway plays a pivotal role in energy metabolism and stress response (38). It is involved in the synthesis of nicotinamide adenine dinucleotide (NAD+), which is essential for cellular energy metabolism, DNA repair, and the regulation of apoptosis, autophagy, and mitophagy. In the TME, mitochondrial autophagy aids tumor cells in surviving stressful conditions, such as hypoxia or nutrient scarcity (39, 40). The VISFATIN signaling pathway influences mitochondrial function and stimulates mitochondrial autophagy by modulating NAD+ levels. Enhanced mitochondrial autophagy enables cells to adapt to metabolic stress, avoid apoptosis, and sustain a tumor-promoting microenvironment. Furthermore, the VISFATIN pathway also impacts the behavior of immune cells, particularly T cells, within the TME. The proper activation and function of T cells rely on the energy supply provided by mitochondria. Dysregulation of mitochondrial autophagy can alter the metabolic state of T cells, resulting in T-cell exhaustion or impaired activation. This T-cell dysfunction is prevalent in the TME, leading to a reduction in tumor immune surveillance and allowing tumor cells to evade immune system detection. Moreover, mitophagy-associated CD8+ T cells exhibit diverse levels of T-cell activity and display inactive features. Specifically, SQSTM1- and MAP1LC3B-expressing CD8+ T cells demonstrate robust activation of the mitophagy and autophagy pathways, accompanied by the upregulation of immune inhibitory molecules, and are characterized as the CD8_exhau subtype. Notably, CD8 exhaustion T cells have conventionally been linked to deleterious effects on antitumor immune responses (41, 42), and our TIDE results further confirmed this point. Interestingly, SQSTM1+ CD8+ cells were beneficial for HCC survival. The abundant expression of immune stimulators may explain this. These findings highlight the critical role of mitophagy within macrophages and T cells in coordinating immune escape and the inherent tumor-promoting potential. Our research demonstrates the complex interactions between mitophagy, HCC immune cell dynamics, and the TME. These multifaceted interactions within the immune–tumor interface may guide cancer treatment strategies.

In our analysis of B cells, we observed intriguing findings. KEGG enrichment indicated the activation of the ribosomal pathway in TOMM20-B-C1 and the IL-17 signaling pathway in VDAC1-B-C3. TOMM20, a crucial component of the mitochondrial outer membrane transport complex, primarily facilitates protein import into the mitochondria (43). The activation of the ribosomal pathway suggests that this cluster may be engaged in heightened protein synthesis, aligning with the high metabolic state observed within the TME. Furthermore, the activation of this pathway might be particularly advantageous for the process of mitophagy in this cluster, offering further insights into the cluster’s role in tumorigenesis and development. Additionally, VDAC1, an essential channel protein on the mitochondrial outer membrane, regulates the exchange of materials between the mitochondria and the cytoplasm (44). Elevated VDAC1 expression often correlates with alterations in mitochondrial function, encompassing oxidative stress and metabolic reprogramming. The IL-17 signaling pathway is intimately linked to inflammation and immune responses, and its activation may signify ongoing immune or inflammatory processes within the TME (45). By influencing metabolism and apoptosis, VDAC1 may participate in tumor cells’ adaptation to the IL-17-induced inflammatory milieu, potentially advancing tumor progression and facilitating immune evasion.

To delineate cell-specific gene regulatory networks, we conducted a single-cell analysis of TFs. Overall, each subtype of CAFs, macrophages, B cells, and CD8+ T cells exhibited a unique TF profile. TOMM7-CAFs exhibit a unique TF gene signature, including FOXN3, ETS2, MYC, FOXP1, NFIB, KLF9, ELF2, JUNB, and TAF7. In addition, better patient survival rates can be observed in this CAF type, which may be due to the unique activated TFs (46, 47). Moreover, for CD8+ T cells, we observed some TFs with expression characteristics similar to those of JUNB cells. Consequently, it is reasonable to hypothesize that mitophagy-associated cell subtypes influence distinct transcription factor regulatory networks to remodel and reprogram the TME. Furthermore, cell network analysis revealed robust connectivity and communication between mitophagy-associated TME and tumor cells. Remarkably, both mitophagic CAFs and immune cell subtypes exhibited heightened communication with cancer endothelial cells, suggesting that TME regulation, including immunosuppression, may be partially influenced by mitophagy.

Considering the intricate inherent patterns of mitophagy within TME cells, we synthesized the associations of these subcluster scores with prognosis and immune response using publicly available bulk RNA-seq datasets. Our analysis revealed substantial prognostic differences among patients with HCC based on the varying dominance of mitophagy-related genes within TME cells. However, distinct mitophagy subtypes do not exhibit exceptional efficacy in response to immune checkpoint blockade (ICB) therapy. This may explain the limited efficacy of immunotherapy in patients with HCC.

Based on our findings, we found that mitophagy markedly affects tumor progression and the prognosis of patients with HCC. However, mitophagy does not appear to impact the effectiveness of immunotherapy. Based on this phenomenon, treatment can be reasonably selected for patients with HCC, and medical waste caused by ineffective immunotherapy can be avoided. Furthermore, advances in interdisciplinary research may lead to more effective adjuvant therapies, potentially addressing the limitations of current immunotherapy approaches. This pioneering treatment approach may benefit a large number of patients with HCC. Although our findings provide valuable insights into the potential association between mitophagy patterns and ICB treatment response, it is important to gain a deeper understanding of the underlying mechanisms. Future studies should focus on mechanistic studies, such as *in vivo* induction or inhibition of mitochondrial autophagy, to directly assess their impact on ICB efficacy. These experiments were critical for validating our observations and determining whether regulating mitochondrial autophagy is a viable strategy for enhancing ICB therapy in HCC.

## 5 Conclusion

Through scRNA analysis, we identified the intrinsic mitophagic cell subtypes within the TME and revealed their involvement in intercellular communication, regulating both tumor growth and antitumor immunomodulation. Our findings emphasize the impact of diverse cell mitophagy models on the prognosis of patients with HCC. Interventions targeting mitophagy may be promising for improving the long-term prognosis of patients with HCC. Our findings provide valuable insights for assessing the prognosis of patients with HCC and offers potential avenues for clinical diagnosis and immunotherapy.

## Data Availability

Publicly available datasets were analyzed in this study. This data can be found here: The datasets mentioned during the current study are available in the GEO database and UCSC Xena database.
