# Supplementary material for: Single-cell mitophagy patterns within the tumor microenvironment modulate intercellular communication, impacting the progression and prognosis of hepatocellular carcinoma
Source: Front Immunol. 2025 Jan 6;15:1448878. doi: 10.3389/fimmu.2024.1448878 (PMC11742944; doi:10.3389/fimmu.2024.1448878)
Supplement: Supplementary file 1 [file DataSheet1.docx]

Supplementary Material

# Supplementary Figures


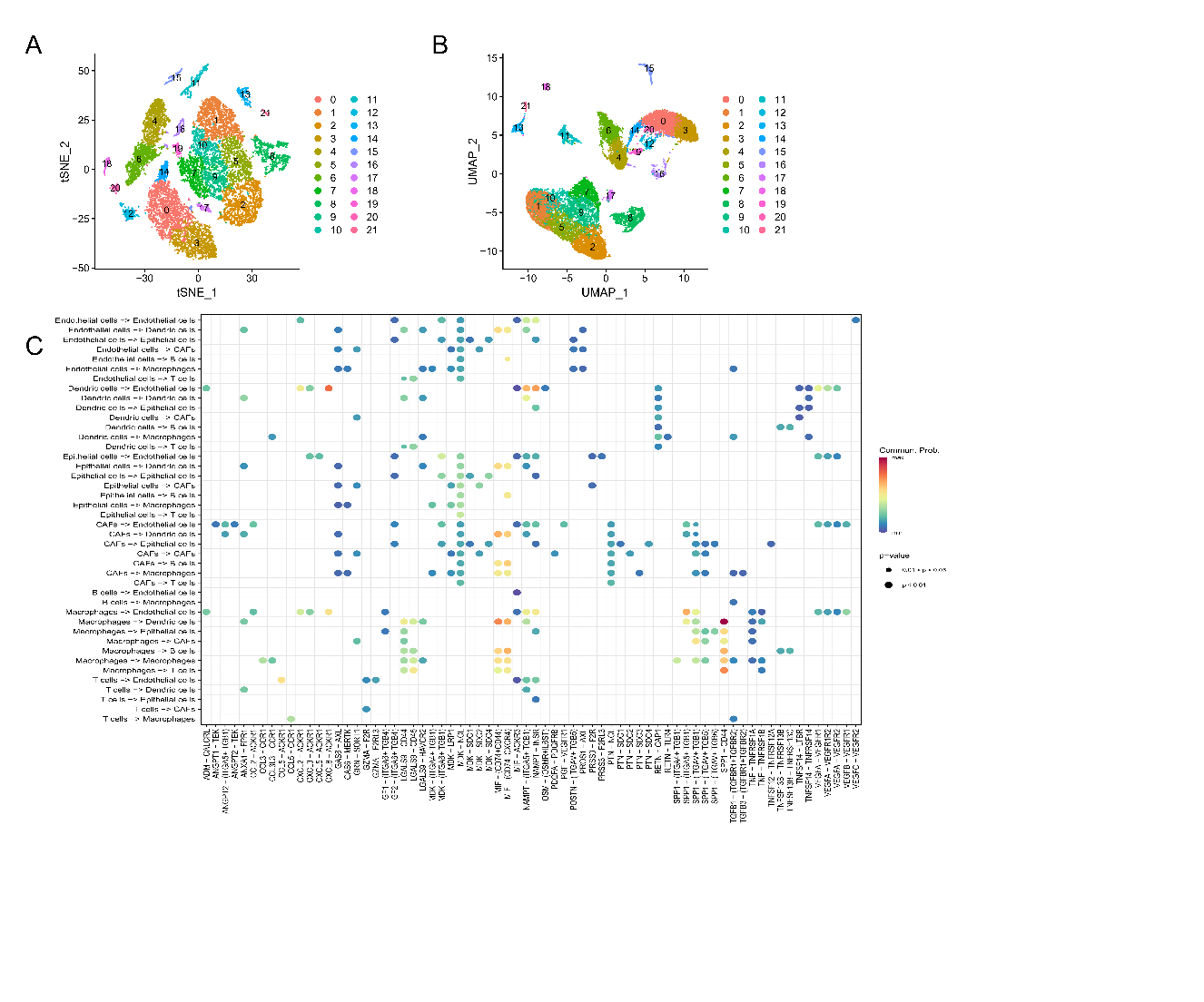


**Supplementary Figure 1. Exploration of HCC cells.**

(A) & (B) UMAP and tSNE plot displaying all HCC clusters. (C) Ligand-receptor interactions among all HCC cell types.


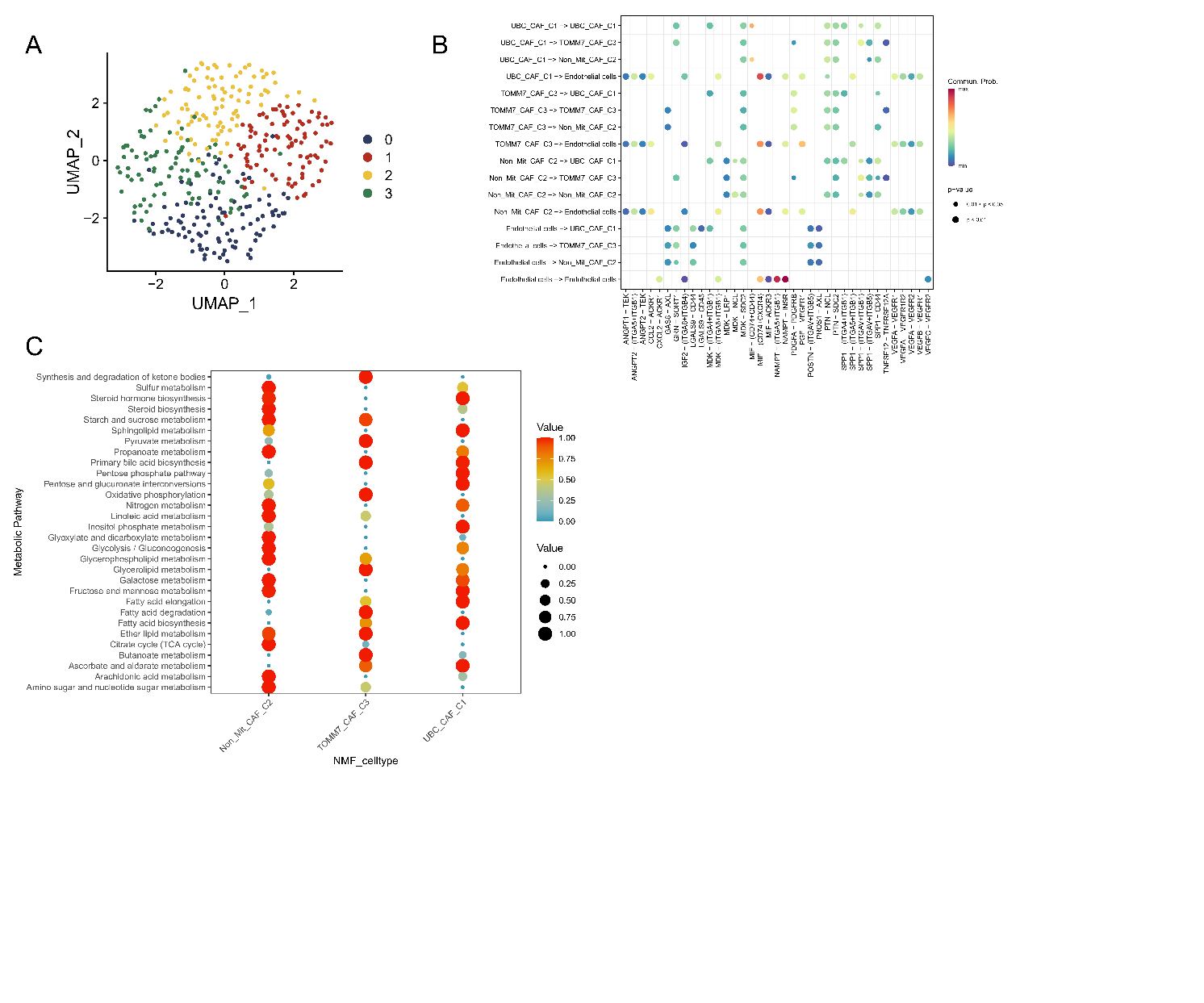


**Supplementary Figure 2. Features of mitophagy-related clusters in CAFs.**

(A) UMAP plot of NMF CAFs clusters. (B) Ligand-receptor interactions among CAFs subtypes and endothelial cells. (C) Metabolic analysis of different NMF CAFs subtypes.


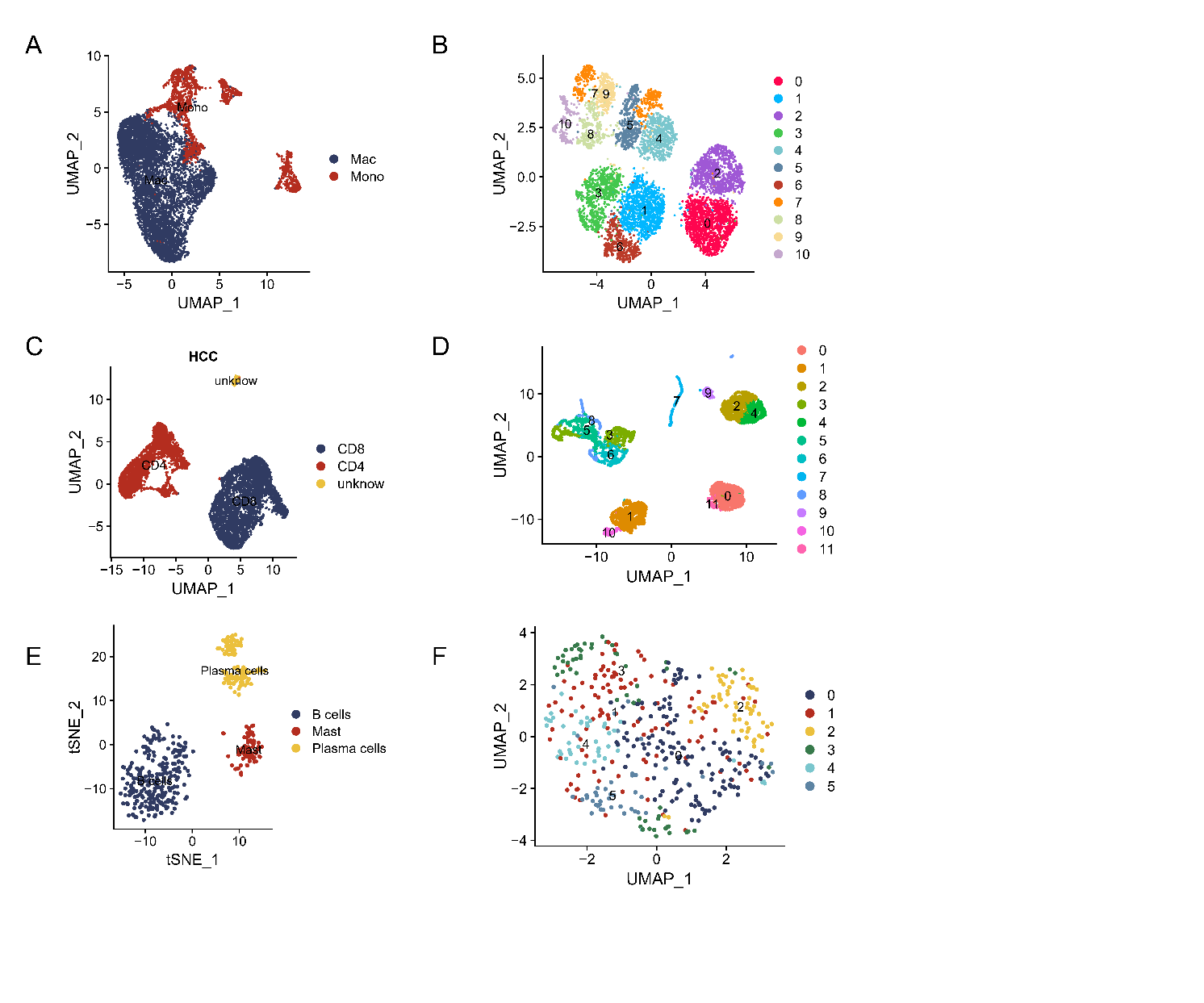


**Supplementary Figure 3. Characteristics of mitophagy-related macrophage.**

(A) UMAP plot of myeloid cells subtypes. (B) UMAP plot of NMF TAMs clusters. (C) UMAP plot of T cells subtypes. (D) UMAP plot of NMF CD8 T cell clusters. (E) UMAP plot of B cells subtypes. (F) UMAP plot of NMF B cell clusters.


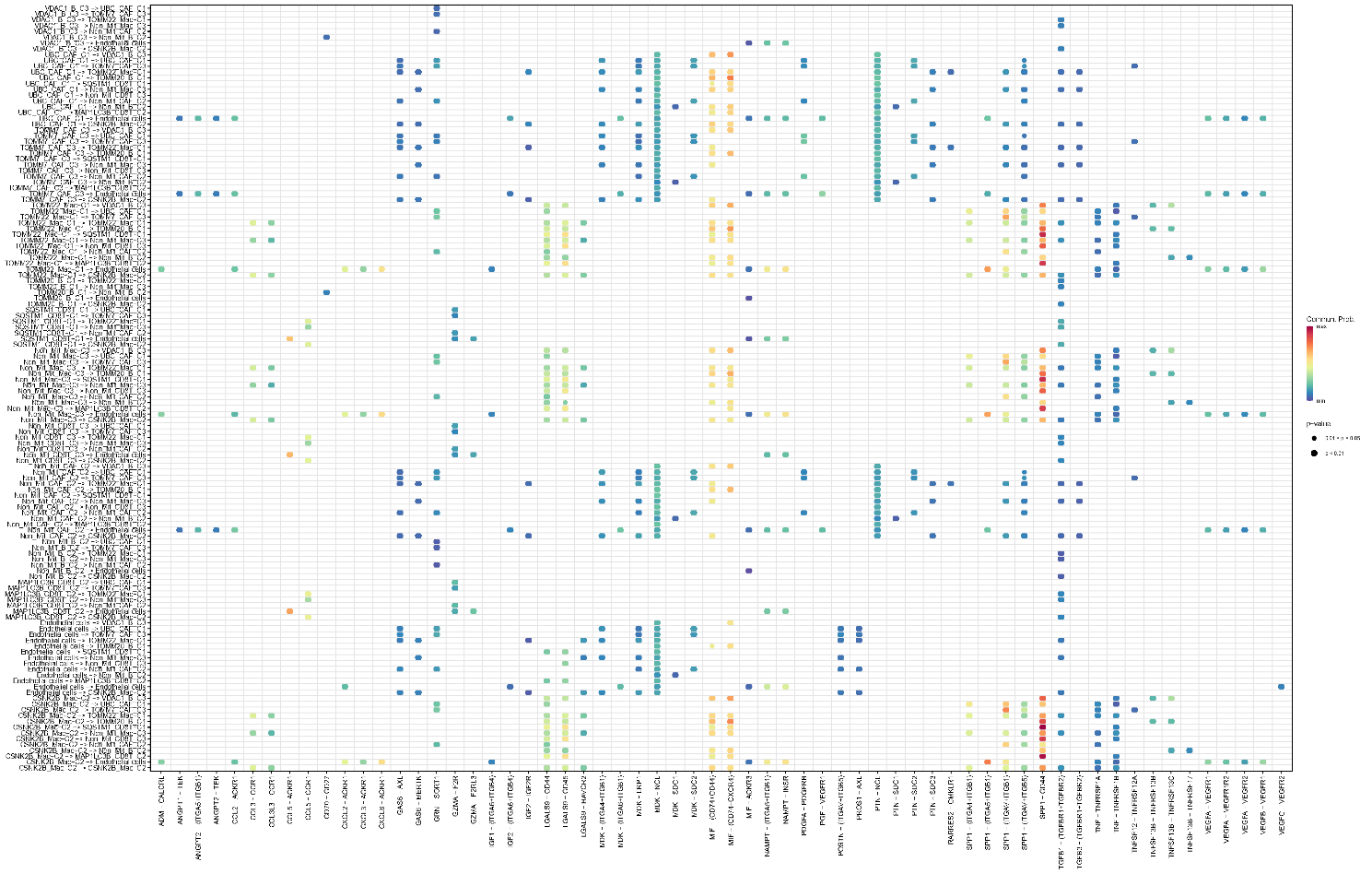


**Supplementary Figure 4. Mitophagy-related analysis and interaction. Ligand-receptor interactions among all mitophagy related subtypes.**

# Supplementary Data

Supplementary Data 1. MITOPHAGY genes.

Supplementary Data 2. CAF subtypes.
